# Impact of psychological factors on the final clinical outcomes of patients undergoing ankle arthrodesis and ankle replacement

**DOI:** 10.3389/fpsyt.2025.1550465

**Published:** 2025-03-06

**Authors:** Hongze Wang, Shihang Cao, Geng Liu, Jun Lu, Junkui Xu

**Affiliations:** Honghui Hospital, Xi’an Jiaotong University, Xi’an, Shaanxi, China

**Keywords:** end-stage ankle arthritis, ankle arthrodesis, ankle arthroplasty, psychological factors, prognosis

## Abstract

**Objective:**

This study aims to analyze the impact of different surgical procedures on the prognosis and psychological state of patients with end-stage ankle arthritis (ESAA) by comparing two groups of patients with ESAA who have undergone total ankle replacement (TAR) and ankle arthrodesis (AA), and to investigate whether preoperative psychological status can alter the final clinical outcomes.

**Methods:**

This study retrospectively collected data from 66 patients with ESAA who underwent AA surgery in the Foot and Ankle Surgery Department of Xi’an Honghui Hospital between 2016 and 2023. In July 2024, the final follow-up of patients was conducted via telephone or WeChat, with a follow-up duration of no less than 12 months. Before surgery and at the final follow-up, evaluations were conducted using the Chinese version of the Hospital Anxiety and Depression Scale (HADS), the Visual Analogue Scale (VAS) for pain (ranging from 0 to 100mm), and the American Orthopaedic Foot and Ankle Society (AOFAS) ankle and hindfoot score. The study compared differences in pain scores, functional scores, and psychological scores between patients in the TAR and AA groups before surgery and at the final follow-up. Additionally, patients who underwent TAR and AA were further subgrouped based on the severity of their preoperative psychological status, in order to analyze the impact of preoperative psychological conditions on surgical prognosis.

**Results:**

A total of 66 patients with ESAA completed the follow-up. At the final follow-up, both the VAS and AOFAS scores in the TAR group and the AA group showed significant improvement compared to preoperative levels. Among them, the TAR group performed better in terms of AOFAS scores, but no significant difference was observed in VAS scores between the two groups. Additionally, there was no significant difference in HADS scores between the two groups at the final follow-up. Regardless of whether they belonged to the high-HADS group or the low-HADS group, patients showed significant improvement in clinical scores compared to preoperative levels. However, at the final follow-up, the clinical scores of the high-HADS group were significantly lower than those of the low-HADS group, and the incidence of complications in the high-HADS group was also higher.

**Conclusion:**

This study found that both TAR and AA significantly improved patients’ psychology, pain, and functional activities. Both surgical methods demonstrated similar improvements in terms of final psychological status and pain relief. However, patients in the TAR group showed better ankle function and mobility. Patients with poorer preoperative psychological status had worse clinical outcomes and faced a higher risk of complications. The study indicates that both TAR and AA are effective treatment options for patients with ESAA, but poor preoperative psychological status is one of the important risk factors for poor prognosis. Therefore, when selecting a treatment approach, the patient’s psychological state and needs should be fully considered, and necessary psychological interventions and postoperative rehabilitation plans should be implemented to enhance the patient’s treatment outcomes and quality of life.

## Introduction

End-Stage Ankle Arthritis (ESAA) is a severe stage of ankle arthritis, similar to end-stage hip arthritis, characterized by severely limited joint function and a very high disability rate (1). According to statistics, patients with ankle arthritis account for approximately 1% of the adult population worldwide and 2% to 4% of all osteoarthritis patients (2).Ankle degenerative disease is often intimately associated with prior trauma (3). Furthermore, it can also be caused by inflammatory joint diseases, hemochromatosis, and hemophilic arthropathy (4).Ankle Arthrodesis (AA) is considered the gold standard for the treatment of ESAA (5). With the continuous updating and improvement of ankle joint implants, the clinical outcomes of total ankle replacement (TAR) have also been significantly enhanced (6–8). Compared with patients undergoing Ankle Arthrodesis (AA), those receiving Total Ankle Replacement (TAR) exhibit better joint function and lower postoperative pain, although there is no significant difference in quality of life between the two surgical procedures (9–13). Furthermore, a meta-analysis study found that TAR has greater short-term advantages compared to AA, but in the medium to long term, TAR becomes disadvantageous due to higher complication and revision rates (14).

The high disability rate and corresponding decrement in quality of life attributable to ankle arthritis inevitably exert an impact on patients’ psychological status (15–17). Numerous scholarly studies have unveiled that psychosocial impairments adversely influence the outcomes and satisfaction associated with other orthopedic surgeries, particularly in the management of degenerative osteoarthritis (18–27). Studies have shown that patients with poorer preoperative psychological status tend to exhibit poorer prognosis and satisfaction after surgery, along with higher pain sensitivity (28) and a greater incidence of postoperative complications (29). However, the current psychological research targeting patients with ESAA appears relatively limited, primarily focusing on the field of TAR, while specific psychological studies on patients with AA are relatively scarce (30–34). This current state significantly restricts surgeons’ comprehensive and in-depth understanding of the psychological needs of this patient population.

Given the current research progress on psychological factors in ESAA surgery, we aim to clarify the changes in psychological status in terms of screening for anxiety, depression, as well as changes in clinical outcome as assessment of pain and ankle function following TAR and AA surgery. Furthermore, we hope to determine whether preoperative poorer psychological status has differential impacts on prognosis, thereby providing a reference for clinicians in devising treatment plans.

## Patients and methods

### Patients

This study was approved by the Ethics Review Committee (No: 202409007). The subjects were all ESAA patients who underwent TAR and AA surgeries in the Foot and Ankle Comprehensive Surgery Department of Xi’an Honghui Hospital between 2016 and 2023. Through case retrieval, it was ensured that all ESAA patients were undergoing TAR or AA for the first time and had a preoperative American Society of Anaesthesiologists (ASA) classification of I-II. In July 2024, the final follow-up of patients was conducted via telephone or WeChat, with a follow-up duration of no less than 12 months. Informed consent was obtained from all patients. Patients were excluded if they had previously undergone tibiotalar-calcaneal arthrodesis, if the surgery was intended for revision of a previous total ankle arthroplasty or ankle arthrodesis, if they had postoperative bone nonunion of the ankle due to previous trauma, if they were unable to communicate normally due to illness. The surgeries were performed by senior surgeons within our department, all of whom had similar levels of experience in both TAR and AA surgeries.

### Outcome measures

We collected demographic data from the patients and assessed their pain and overall functional status using the American Orthopaedic Foot and Ankle Society (AOFAS) Ankle-Hindfoot Scale and the Visual Analogue Scale (VAS) for pain both preoperatively and at the last follow-up. The Chinese Version of the Hospital Anxiety and Depression Scale (HADS) was used to investigate patients’ anxiety and depression levels both preoperatively and at the last follow-up.

The AOFAS Ankle-Hindfoot Scale, with a maximum score of 100 points, comprehensively and objectively assesses the patient’s ankle condition by encompassing pain (40%), function (50%), gait (10%), and other related indicators (35). The VAS is currently widely employed for pain assessment. It comprises a 100mm line, with one end denoting “no pain at all,” labeled as “0,” and the other end representing “the worst pain imaginable” or “pain at its most intense level,” labeled as “10.” Patients are instructed to mark a point on the line according to their own perception of pain (36). The HADS (37) consists of two subscales: Anxiety (HADS-A) and Depression (HADS-D). It includes seven items for assessing depression and seven items for assessing anxiety. There are six reverse-scored items in total, with five in the Depression subscale and one in the Anxiety subscale. Each item is rated on a 4-point scale ranging from 0 to 3, resulting in a total score range of 0 to 21. Scores of 0 to 7 indicate no symptoms, 8 to 10 suggest possible anxiety or depression, and 11 to 21 confirm the presence of anxiety or depression. We categorize scores of 8 or above as the high-HADS group (indicating a high risk of anxiety/depressive state), and scores below 8 as the low-HADS group (indicating a low risk of anxiety/depressive state) (37).

We first divided the patients into the TAR group and the AA group, and then further subclassified each surgical group into a low-HADS group (indicating a preoperative low risk of anxiety/depressive state) and a high-HADS group (indicating a preoperative high risk of anxiety/depressive state). We grouped patients according to their surgical techniques and preoperative psychological states, aiming not only to more intuitively reflect the differences in surgical outcomes between the two techniques, but also to ascertain whether preoperative psychological states have an impact on the prognosis of each surgical technique.

### Statistical analysis

We reported the demographic information of the patient population and classified the patients into the TAR group and the AA group. Furthermore, each subgroup was further subdivided into the low-HADS group and the high-HADS group. Statistical analysis of the data was performed using SPSS software version 27.0 (IBM, New York, United States).Firstly, the Shapiro-Wilk test was used to assess the normality of the data distribution, in order to determine whether parametric or non-parametric tests should be employed. For measurement data that satisfy normal distribution, they are expressed as mean ± standard deviation (x ± s), and comparisons between two groups are conducted using the independent samples t-test, with paired t-test applied to paired measurement data. For measurement data that do not satisfy normal distribution, they are presented as M(P25, P75), and comparisons between two groups are performed using the Mann-Whitney U test, with the Wilcoxon Signed Rank Test applied to paired measurement data. For count data, they are represented as n(%), and comparisons between two groups of measurement data are conducted using the chi-square test. Baseline characteristics were compared using the independent samples t-test, Mann-Whitney U test, and chi-square test. Differences in preoperative and postoperative evaluation indicators were primarily analyzed using the independent samples t-test or Mann-Whitney U test. To evaluate the differences in various evaluation indicators between preoperative and last follow-up within the TAR and AA groups, we used the paired samples t-test or Wilcoxon Signed Rank Test. For the differences in preoperative and final clinical outcomes between the low HADS group and high HADS group within the same subgroup, we also conducted analysis using the independent samples t-test or Mann-Whitney U test. The significance level for statistical tests was set at P < 0.05.

## Results

In our study, a total of 100 patients with eligible ESAA were enrolled, among which 66 patients completed the follow-up, resulting in a loss to follow-up rate of 44%. The most common reasons for loss to follow-up were change of contact information and refusal to participate in the final follow-up. Of the 66 patients who completed the follow-up, 30 were in the TAR group and 36 were in the AA group. The mean age of the patients was 57.56 ± 10.16 years, ranging from a minimum of 27 years to a maximum of 77 years. The average follow-up time for all patients was 34.35 ± 22.04 months, with the shortest follow-up being 12 months and the longest being 88 months. Preoperatively, 25 patients (37.88%) had high HADS scores. In the TAR group, 2 patients (6.7%) experienced postoperative osteophyte formation and 1 patient (3.3%) had prosthesis subsidence. In the AA group, 2 patients (5.6%) developed bone nonunion and 1 patient (2.8%) had poor wound healing. Baseline characteristics of the two patient groups are presented in **Table 1**.

**Table 1:** Demographic characteristics of patients undergoing TAR and AA.

	Total (n = 66)	Group TAR (n=30)	Group AA (n=36)
Sex			
Male	20 (29.9%)	8 (25.8%)	12 (33.3%)
Female	46 (68.7%)	22 (71%)	24 (66.7%)
BMI (kg/m^2^)	23.34 ± 3.15	23.30 ± 3.09	23.38 ± 3.23
Age	57.56 ± 10.16	55.67 ± 11.08	59.14 ± 9.18
Follow-up duration (Month)	34.35 ± 22.04	36.10 ± 27.75	32.89 ± 16.10
Complication			
Osteophytosis	2 (3.0%)	2 (6.7%)	0
Implant Subsidence	1 (1.5%)	1 (3.3%)	0
Nonunion	2 (3.0%)	0	2 (5.6%)
Impaired wound healing	1 (1.5%)	0	1 (2.8%)

There were no significant differences in the baseline preoperative scores between the TAR and AA groups (P > 0.05, **Table 2**). At the final follow-up, both groups demonstrated substantial improvements in their clinical outcomes compared to the preoperative state (p>0.05, **Table 3**). The TAR group showed superiority only in the AOFAS score, with a mean of 80.47 ± 12.56, compared to the AA group’s mean of 73.28 ± 8.81 (p<0.01, **Table 2**). However, there were no statistically significant differences in either the VAS score or the psychological score (HADS) between the two groups (P>0.05, **Table 2**). This suggests that both surgical approaches provide similar improvements in pain relief for ESAA, but TAR offers better ankle mobility.

**Table 2:** Differences in baseline and final follow-up clinical outcome scores between the TAR and AA groups overall.

	Group TAR	Group AA	t/z Value	P Value
Preoperative				
VAS	6.71±0.42	6.72±0.40	-0.153	0.879
AOFAS	36.00 (30.00,46.25)	34.00 (26.00,42.00)	-1.775	0.076
HADS-A	5.00 (3.00,9.00)	6.64±2.60	-4.604	0.249
HADS-D	4.00 (2.00,9.00)	6.22±2.79	-4.587	0.309
Postoperative				
VAS	1.55 (1.20,3.20)	2.20 (1.80,2.60)	-3.223	0.085
AOFAS	81.00 (75.00,89.00)	73.28±8.81	-3.136	<0.01
HADS-A	3.00 (2.00,6.00)	3.00 (2.00,5.00)	-4.285	0.953
HADS-D	3.00 (1.00,8.00)	3.00 (1.00,4.50)	-4.446	0.600

**Table 3:** Comparison of baseline and final follow-up clinical outcome scores within the TAR and AA groups.

	Group TAR		Group AA	
Score Comparison	z	P-value	z	P-value
Baseline VAS VS Last follwo-up VAS	-4.763	<0.001	-5.236	<0.001
Baseline AOFAS VS Last follwo-up AOFAS	-4.784	<0.001	-5.233	<0.001
Baseline HADS-A VS Last follwo-up HADS-A	-4.236	<0.001	-5.198	<0.001
Baseline HADS-D VS Last follwo-up HADS-D	-3.943	<0.001	-5.105	<0.001

In both the TAR and AA groups, regardless of whether the patients belonged to the low HADS group or the high HADS group preoperatively, their final clinical scores showed significant improvements compared to their preoperative baseline scores (p < 0.05), as shown in **Table 4**.

**Table 4:** Differences in improvement of final clinical outcome scores based on preoperative psychological status in the TAR and AA groups.

	Group TAR				Group AA			
	Low HADS	High HADS	t/z/x^2^Value	P Value	Low HADS	High HADS	t/z/x^2^Value	P Value
Baseline VAS	6.61 ± 0.39	6.85 ± 0.44	-1.557	0.131	6.63 ± 0.41	6.89 ± 0.34	-1.894	0.067
Last follow-up VAS	1.41 ± 0.48	3.42 ± 1.75	-4.652	<0.001	2.00 ± 0.45	2.95 ± 0.88	-4.263	<0.001
Baseline AOFAS	36.00(27.00,46.00)	38.67 ± 9.57	-0.702	0.482	35.61 ± 7.43	27.00(23.00,40.00)	-1.821	0.069
Last follow-up AOFAS	85.94 ± 6.54	75.00(70.00,77.50)	-3.136	0.020	76.39 ± 6.89	67.77 ± 9.38	3.160	0.003
LOS	13.00(11.00,14.00)	13.33 ± 3.96	-0.299	0.765	8.00(7.00,9.00)	10.31 ± 3.22	-1.931	0.053
Complication	0	3	2.608	0.025	0	3	3.163	0.016

Among patients undergoing TAR and AA, there were no significant differences in preoperative VAS scores and preoperative AOFAS scores between the low-HADS group and the high-HADS group (p > 0.05). However, among patients in the preoperative high-HADS group, those who underwent TAR and AA had significantly lower VAS scores and AOFAS scores at the final follow-up compared to patients in the preoperative low-HADS group (p < 0.05). There was no significant difference in the length of stay (LOS) between the low-HADS group and the high-HADS group (p > 0.05). In terms of postoperative complications, the incidence was significantly higher in the high-HADS group compared to the low-HADS group (P < 0.05), as shown in **Table 4**.

## Discussion

To determine whether the better range of motion observed postoperatively in TAR compared to AA can lead to better psychological improvement for patients, we compared the final clinical outcomes and psychological scores between TAR and AA. There is robust evidence that emotional disorders such as depression and anxiety have significant negative impacts on the prognosis of patients with knee and hip arthritis (18–27), but research in the field of foot and ankle surgery is still scarce. Given the significant differences in etiology, functional impairment, and clinical manifestations between ESAA and other end-stage arthritides, this specificity may exert varying degrees of influence on an individual’s psychological state, pain perception, and daily function. Therefore, we further investigated the differences in final clinical outcomes between ESAA patients with preoperative high-risk and low-risk anxiety/depression states who underwent TAR and AA treatment, in order to comprehensively assess the impact of psychological status on treatment efficacy.

At the final follow-up, patients who underwent either TAR or AA reported significant improvements in outcome indicators compared to their preoperative status. However, patients with high HADS scores reported more severe pain and poorer function at the final follow-up compared to those with low preoperative HADS scores. A large number of previous studies have clearly demonstrated that psychological distress is an important risk factor for poor surgical outcomes in patients with musculoskeletal diseases. It not only adversely affects the degree of postoperative pain but also significantly reduces patients’ functional recovery and overall satisfaction (23–27, 38). Cunningham et al. conducted a correlation analysis of risk factors affecting outcomes after TAR surgery and found that depression had a negative effect on patient-reported outcomes (32). In a recent study by Cunningham et al., preoperative mental health status and depression were analyzed as risk factors, and it was found that although the clinical outcomes of all patients improved compared to the preoperative status, the improvement in final outcomes was relatively poorer among patients with preoperative depression and poorer mental health (33). Kim et al. used the Center for Epidemiologic Studies Depression Scale (CES-D) and the Patient Health Questionnaire-9 (PHQ-9) to assess preoperative depression status in TAR patients. The study found that patients with depression had lower final clinical indicators compared to those without depression (34). However, it is noteworthy that there were no statistically significant differences in the two key indicators, preoperative VAS scores and AOFAS scores, between the two groups (34), which is consistent with our analysis results.

Our study also found that regardless of whether patients had poor or good preoperative psychological status, their psychological state significantly improved after undergoing TAR and AA surgical interventions. This finding suggests that preoperative anxiety and depression are largely reversible, and this positive psychological transformation is likely directly related to the effective surgical intervention. However, at the final follow-up stage, despite patients in the TAR group showing a significant advantage in terms of joint range of motion compared to the AA group, there was no statistically significant difference between the two groups in the two psychological assessment dimensions of HADS-A and HADS-D. Drawing upon previous research findings, we speculate that the absence of statistically significant differences in psychological scores may be associated with the equivalent performance of TAR and AA in postoperative pain relief, gait recovery, and overall patient satisfaction (14, 39–42). Nevertheless, we must recognize that psychological states can be influenced by various factors (43), necessitating further in-depth research in the future to investigate this matter.

At the final follow-up, no complications were observed in the low-HADS groups of both the TAR and AA groups. However, in the TAR group, two patients with high-HADS scores before surgery developed osteophyte hyperplasia accompanied by pain, and underwent a second joint debridement surgery. In addition, one more patient suffered from abnormal limb alignment as a result of prosthesis subsidence. In the AA group, two patients with preoperative high-HADS group had nonunion and one had poor wound healing. These results indicate that poor preoperative psychological status may increase the risk of postoperative complications, which is consistent with the findings of Harmer’s research in the hip and knee joint areas (29). Simultaneously, we observed that preoperative psychological status did not significantly affect patients’ LOS. This finding is consistent with the results of Sutherland et al.’s study on risk factors for ESAA surgery (44). This indicates that preoperative psychological status may influence the incidence of postoperative complications, but its impact on the LOS may be relatively minor, and LOS may be influenced by other factors. Therefore, conducting preoperative psychological assessments and providing appropriate interventions for patients have positive implications for reducing the risk of postoperative complications. However, the management of LOS requires comprehensive consideration of multiple factors for optimization.

In this study, patients undergoing ESAA were self-assessed using the HADS upon admission to evaluate their preoperative psychological state, which generally results in less observer bias. Furthermore, the use of appropriate scale tools can improve time and cost benefits, making it feasible for routine application in orthopedic clinical practice. By thoroughly obtaining and analyzing the patient’s clinical history and conducting systematic mental health screenings, once a patient is identified with higher psychological risks, they should be promptly referred to professional mental health experts, and based on this, a targeted and personalized exercise and rehabilitation plan should be developed. By taking such measures, surgeons can more effectively mitigate the risk of adverse clinical prognosis that patients may face due to psychological issues, thereby providing solid support and assurance for the patient’s overall rehabilitation process.

This study has several limitations. Firstly, regional bias and sample size constraints may affect the generalizability of the results, and we still require large-sample, multicenter studies to further validate the conclusions of this study. Secondly, the generalization of psychological assessment tools also needs to be considered, and interdisciplinary teams are required in the later stages to deepen psychological assessment and treatment planning. Finally, the retrospective design, accompanied by high loss to follow-up rates and retrospective bias, may lead to biased results. It is recommended that future studies adopt randomized controlled trials to enhance scientific rigor and objectivity. These limitations should be fully considered when interpreting and generalizing the results of this study to ensure the reliability, validity, and broad applicability of the research conclusions.

## Conclusion

We found that both TAR and AA significantly improved patients’ psychological well-being, pain, and functional activities, with similar improvements in final psychological status and pain relief. In terms of ankle range of motion, TAR was superior to AA. However, patients with poorer preoperative psychological status had significantly worse clinical outcomes and a higher risk of complications compared to those with better preoperative psychological status. This study indicates that both TAR and AA are effective treatment options for ESAA, but poorer preoperative psychological status is one of the risk factors for poor prognosis after TAR and AA surgeries. Therefore, when selecting a treatment approach, the patient’s psychological state and needs should be fully considered, and necessary psychological interventions and postoperative rehabilitation plans should be implemented to enhance the patient’s treatment outcomes and quality of life.

## Data Availability

The raw data supporting the conclusions of this article will be made available by the authors, without undue reservation.
